# A diagnostic headache: A rare case of adult onset primary leptomeningeal medulloblastoma

**DOI:** 10.1177/2050313X261459345

**Published:** 2026-06-29

**Authors:** Sophia Raefsky, Neal Nathan, Meshari Alharthi, Jessica Bloom, Vanessa Goodwill, Kari Hird, Nikdokht Fari, William Ueng, Jessica Schulte

**Affiliations:** 1 Department of Neurosciences, University of California, San Diego, La Jolla, CA, USA; 2 Department of Internal Medicine, Umm-Alqura University, Makkah, Saudi Arabia; 3 Department of Pathology, University of California, San Diego, La Jolla, CA, USA; 4 Department of Pathology, University of Utah, Salt Lake City, UT, USA; 5 Department of Radiology, University of California, San Diego, La Jolla, CA, USA; 6 Department of Neurology, Torrance Memorial Medical Center, La Jolla, CA, USA; 7 Department of Neurosciences, New York University, NY, USA

**Keywords:** medulloblastoma, leptomeningeal disease, headache

## Abstract

Headache is a common reason for consulting inpatient neurology and the most common outpatient reason for referral to neurology clinics. Idiopathic intracranial hypertension is a common cause of secondary chronic headaches. Presence of typical patient traits and comorbid risk factors such as obesity could lead to bias and misdiagnosis without investigation into other etiologies. Primary leptomeningeal medulloblastoma in adults is an extremely rare cause of headache that initially mimicked idiopathic intracranial hypertension in this case. A 25-year-old male with months of intractable headaches developed confusion, vision changes, and weakness. Headaches were initially attributed to idiopathic intracranial hypertension, so spinal imaging was not attained at other hospitals, and he was first treated with cerebrospinal fluid diversion and optic nerve sheath fenestration. However, he later developed decompensated hydrocephalus and myelopathy. Imaging at our hospital showed extensive leptomeningeal enhancement in the brain, spinal cord, and cauda equina. A lumbar intradural mass was biopsied with pathology showing nodular/desmoplastic and anaplastic/large cell medulloblastoma, consistent with disseminated isolated leptomeningeal medulloblastoma. This case highlights that when headaches are refractory, the differential diagnosis should be expanded, and comprehensive work-up should be pursued. Leptomeningeal metastasis is a rare cause of intractable headaches. Further, isolated leptomeningeal medulloblastoma is rare. Early recognition, comprehensive neuroimaging, and prompt tissue biopsy are critical to facilitating an accurate diagnosis and optimizing therapeutic opportunities. Rapid clinical and radiographic response to proton craniospinal irradiation and the Packer protocol suggests this regimen is effective for adult-onset primary leptomeningeal medulloblastoma.

## Introduction

Headache is a common and nonspecific neurological symptom with a wide differential diagnosis. Chronicity, duration of symptoms, quality of headache, associated neurological symptoms, and provoking and palliating factors are all subjective clinical elements that inform a diagnosis. Headache can be primary or secondary in etiology, and important causes to consider are cerebral venous sinus thrombosis, giant cell arteritis, idiopathic intracranial hypertension (IIH), intracranial hypotension, and migraine.^1^ Leptomeningeal disease is an important cause of intractable headache and represents a diagnostic challenge due to its nonspecific clinical presentation and relative rarity. For patients with symptoms like encephalopathy or focal neurological deficits, work-up beyond bedside evaluation should start with a screening computed tomography (CT) brain scan and likely require magnetic resonance imaging (MRI) with contrast. If there is no concern for impending herniation, a lumbar puncture with an opening pressure, should be obtained and cerebrospinal fluid (CSF) studies including infectious labs and cytology should be sent. Patients with chronic headaches often have multiple presentations to various care settings with prior work-up that can bias an evaluation during a new presentation. Each new presentation of headache, especially if a different headache semiology should prompt the provider to re-evaluate if the prior diagnosis is correct and if there are any red flags requiring further workup.

In this case, a 25-year-old male with protracted headaches and extensive prior work-up was diagnosed with IIH. He was admitted to our hospital, with further neurological decline and was found to have diffuse leptomeningeal enhancement, ultimately diagnosed with medulloblastoma. We therefore present an interesting and rare case of disseminated primary leptomeningeal medulloblastoma in a young adult.

## Case presentation

A 25-year-old male with no prior medical conditions, first presented to an outside hospital with severe headaches, vision loss, blurred vision, nausea, and vomiting. His initial workup included a lumbar puncture with elevated opening pressure. Brain MRI/Magnetic Resonance Venography (MRV) was read as a focus of enhancement with restricted diffusion locally superficially on the right cerebellum suggesting a subacute infarct. After this evaluation, he was diagnosed with IIH and started on the carbonic anhydrase inhibitor and diuretic, acetazolamide. He was also started on aspirin 81 mg for the stroke on imaging. He underwent placement of a ventriculoperitoneal shunt (VPS) 3 months after the initial diagnosis of IIH as his headaches continued to persist. When he continued to have headaches and blurry vision despite the shunt, he underwent optic nerve sheath fenestration surgery 4 months after initial presentation. Of note, no spinal images were done as part of prior workup to assess blockage of the CSF which can also cause elevated intracranial pressure.

Five months after initial symptoms, he presented to our hospital with confusion, difficulty speaking and comprehending, severe headache, nausea, vomiting, gait imbalance and arm and leg incoordination, dysmetria on right finger-nose-finger, hand weakness, constipation, and urinary retention. With this constellation of symptoms, there was concern for the development of decompensated hydrocephalus. His MRI brain showed diffuse nodular leptomeningeal enhancement throughout the skull base cisterns and extending to the spinal canal without a parenchymal lesion (Figure 1). On the radiology read, the previously diagnosed right cerebellar stroke was identified as extra-axial enhancement thought to be part of the leptomeningeal process. MRI of the spine showed diffuse, thick, and nodular leptomeningeal enhancement throughout the cranio-caudal extent of the spinal cord and extending into the cauda equina. In addition, MRI of the spine also showed significant cervical cord edema without a clear intrinsic cord lesion suggestive of venous congestion rather than parenchymal mass. There were also L2 and L3 vertebral body-enhancing lesions. Given the chronic course and widespread disease, the differential was wide, including atypical or granulomatous processes such as a fungal infection, tuberculosis, neurosarcoidosis, demyelinating disease, or neoplastic process, such as primary diffuse midline glioma/glioblastoma, primary central nervous system (CNS) lymphoma, or secondary lymphoma. He was started on steroids for his spinal cord intramedullary edema, with rapid improvement in his myelopathic symptoms including hand weakness, urinary retention, and constipation.

**Figure 1.**
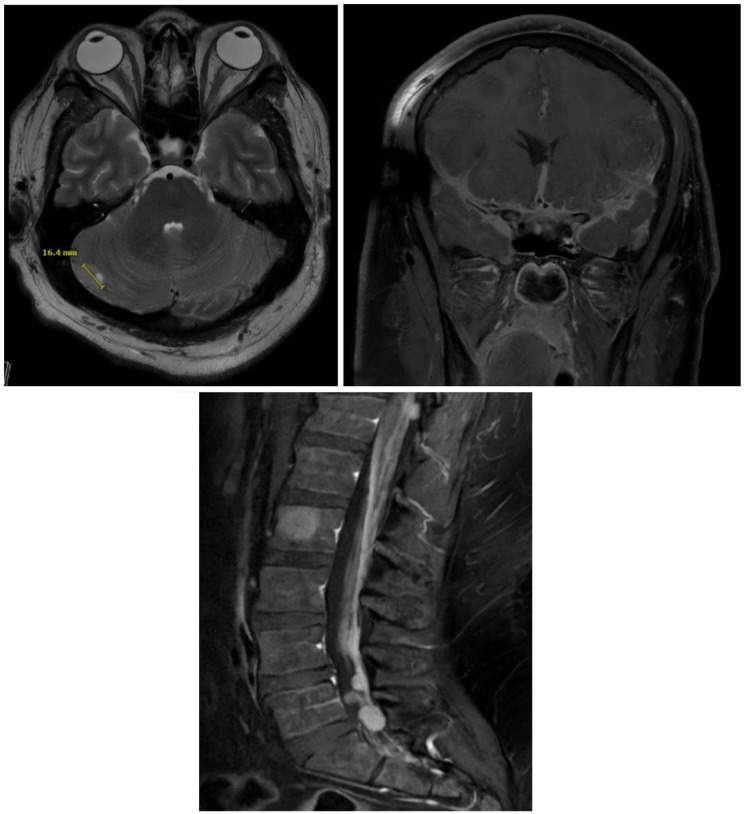
MRI images. The patient had an MRI brain and spine with and without contrast as part of his workup. Imaging showed extensive leptomeningeal enhancement in the brain, including a right cerebellar lesion that when discussed with radiology was thought to be extra-axial. He additionally had lesions throughout the spinal cord and cauda equina. The lesions were all responsive to radiation therapy. MRI: magnetic resonance imaging.

Serum and CSF test results are summarized in Table 1. CSF was sampled from the patient’s shunt, and CSF testing showed WBC 5, RBC 642, Protein 27, glucose 83. Infectious, inflammatory, and autoimmune workup was all negative. This profile is most consistent with a traumatic sample when accessing the VPS given the presence of red blood cells and lack of hemorrhage on multiple brain scans. Eventual CSF cytopathology showed a few clusters of atypical cells as well as scattered multinucleated giant cells and histiocytes. Subsequent CSF flow cytometry was within normal limits (Table 1). A tissue biopsy of one of the larger lesions along the cauda equina showed medulloblastoma with desmoplastic and anaplastic/large cell histology, CNS WHO grade 4. Further immunohistochemical markers such as GAB1 and YAP1 were diffusely positive and testing was consistent with sonic hedgehog (SHH)-activated three subgroup.^2^ His microarray showed loss of heterozygosity of 3p, loss of chromosome 14q and 17p, and 13.3-p12 (which includes TP53). Medulloblastoma can metastasize outside of the CNS, so FDG–PET was performed outpatient which showed a FDG-hyperavid lesion in L2 vertebral body, suggestive of a metastasis.

**Table 1.** Laboratory data.

Variable	Reference range	Lab result
Serum		
HIV 1/2 antibody	Nonreactive	Nonreactive
RPR (Rapid Plasma Reagin)	Nonreactive negative	Nonreactive
*Aspergillus* galactomannan antigen	Negative	Negative
Cryptococcal antigen screen	<1.0000	Negative
Coccidioides antibody lgG, lgM (Enzyme Immunoassay units)	0.34 IU/mL or less	Negative
Quantiferon Gold (IU/mL)	Negative	Negative
Anti-nuclear antibody	0–13.0	Negative
Rheumatoid factor (IU/mL)	Negative	Negative
Anti-neutrophil cytoplasmic Ab	90.0–180.0	142.0
C3 (mg/dL)	10.0–40.0	32.0
C4 (mg/dL)	25.0–175.0	151.0
LDH (Lactate Dehydrogenase) (U/L) lgG subclass 1, 2, 3, 4		Within normal range
Interleukin 2 receptor (pg/mL)	175.3–858.2	240.1
Beta 2 microglobulin (mg/L)	0.8–2.2	1.8
Cytokine 13 panel (pg/mL)		^a^
NMO (Neuromyelitis Optica)/aquaporin-4	Negative	Negative
MOG (Myelin Oligodendrocyte Glycoprotein)	Negative	Negative
Flow cytometry		^b^
CSF		
White blood cells (/mm^3^)	0–5.0	5.0
Red blood cells (/mm^3^)	0	642.0
Glucose (mg/dL)	40.0–70.0	83.0
Protein (mg/dL)	15.0–45.0	27.0
Angiotensin converting enzyme (U/L)	16.0–85 0	16
Cytopathology		A few clusters of atypical cells and scattered multinucleated giant cells and histiocytes
Flow cytometry		No monotypic B-cell population. T-cell CD4:CD8 ratio of 1.5:1, within normal range

CSF: cerebrospinal fluid.

Patient had both serum and CSF studies in the evaluation of his symptoms. His serum testing was negative for the lab values listed below. His CSF did not show signs of any infectious or inflammatory process. His CSF flow cytometry was unremarkable. Cytopathology showed a few clusters of atypical cells as well as scattered multinucleated giant cells and histiocytes.

a Elevated interleukin 10 (20.1, reference range ⩽2.8) and elevated interleukin 6 (4.4, reference range ⩽2). Remainder of cytokines within normal range.

b Zero point zero three percent myeloid blasts with immunophenotypic variations, the significance of which is not known. There are no monotypic B-cells. The T cells exhibit normal CD4:CD8 ratio at 2.9:1. NK cells are not increased.

He underwent proton whole craniospinal irradiation (CSI) including the L2 lesion followed by Packer protocol (with cisplatin, lomustine, and vincristine) for medulloblastoma. He is currently being treated with a year of consolidation therapy with vismodegib, an inhibitor of smoothened, downstream of SHH signaling. He was also treated with bevacizumab for edema. Interval MRI scans after treatment showed improvement in leptomeningeal disease. Treatment was complicated by polyneuropathy attributed to radiation and chemotherapy, and fatigue in the context of low testosterone (Figure 2)).

**Figure 2.**
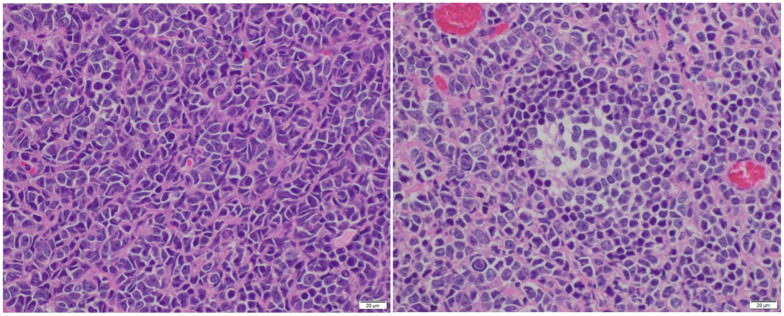
Pathology. H&E stained sections show: On the left large cell/anaplastic morphology characterized by anaplastic pleomorphic nuclei, nuclear molding, and cell-cell wrapping. On the right A central area of desmoplastic/nodular morphology characterized by densely cellular sheets and single files of smaller nuclei interrupted by lucent, less cellular, well-circumscribed pale nodules of cells. Images were taken at 40× magnification.

## Discussion

We present a case of a patient with months of intractable headaches, who was found to have leptomeningeal enhancement on MRI. The time course of chronic headache was also suggestive of an indolent process, and initial CT imaging on presentation ruled out causes of acute headache such as intracerebral hemorrhage or subdural hematoma. In retrospect, there were multiple atypical features about the diagnosis of IIH, including male gender, progressive neurological deficits, and the persistence and severity of symptoms despite standard Intracranial Pressure (ICP) interventions should have triggered a repeat, extended evaluation for possible etiologies of secondary hydrocephalus, including spinal imaging to evaluate for distal causes of elevated ICP. The differential for this patient was broad, including infectious etiologies, especially tuberculosis or fungal etiologies, inflammatory, such as neurosarcoidosis, or neoplastic, related to primary or secondary (metastatic cancer), as well as paraneoplastic (Supplemental Material).

Headache is a nonspecific symptom, and even headache related to elevated intracranial hypertension from leptomeningeal disease has a wide differential of etiologies. Primary leptomeningeal cancer is hard to detect and patients usually present with headache, so diagnosis is usually delayed, and surgical cure is not an option.^3^ Other symptoms more suggestive of possible leptomeningeal involvement include cranial neuropathies such as dysarthria or face weakness, numbness with a sensory level, and cauda equina syndrome with constipation and urinary retention. However, even after diagnosis, the paucity of isolated leptomeningeal solid cancer, especially for medulloblastoma, and the variegated molecular and histological profiles complicate management options.

Primary leptomeningeal medulloblastoma is an exceptionally rare diagnosis. Medulloblastomas are the most common malignant pediatric brain tumor, accounting for 10%–25% of primary CNS tumors in children and 1% in adults, with an incidence of 0.6–1 cases/million/year.^3–5^ The peak incidence in children is between 5 and 9 years old, although it can affect young adults.^4^ Medulloblastomas classically are well-defined masses in the posterior fossa, such as the cerebellum that can have subsequent metastatic spread to the subarachnoid space and leptomeninges.^3^ Primary leptomeningeal medulloblastoma without a primary mass is exceedingly rare, with only a handful of case reports that have only been described in children only, and none described in adults.^4^

Medulloblastoma generally presents with symptoms associated with increased intracranial pressure, such as headaches, nausea, vomiting, and a sign of optic nerve head edema.^6^ The common symptoms are due to posterior fossa location and propensity for hydrocephalus due to obstruction of the fourth ventricle.

The current 2021 WHO classification of tumors of the CNS includes both histological and molecular criteria for medulloblastoma, defining four molecular types (wingless-type (WNT)-activated, SHH activated TP53-wildtype, SHH-activated and TP53-mutant, and non-WNT/non-SHH) and four histological patterns (classic, desmoplastic/nodular, Medulloblastoma (MB) with extensive nodularity, and large cell/anaplastic).^7^ With heterogeneous tumor profiles, there are also heterogeneous outcomes; most data on survival relates to pediatric cases with focal medulloblastoma. SHH-activated is the most common molecular subtype of adult medulloblastoma, comprising 60% of cases with a 5-year overall survival (OS) rate of 50%–70% if patients do not have a concomitant TP53 mutation, which lowers survival to <50%.^6^ However, for cases of SHH-activated medulloblastoma, 10-year progression-free survival (PFS) and OS rates were similar between focal and disseminated medulloblastoma, with PFS 81.2% and OS 93.7%, respectively.^8^ Treatment uniformly includes whole brain and spine radiotherapy, and chemotherapy. In the presence of SHH-alterations, a targeted inhibitor, vismodegib, is also used.

This case is unique for both the presentation of isolated leptomeningeal medulloblastoma in an adult, and the delay in diagnosis. The postulated theory for primary leptomeningeal disease without primary mass is that it is thought to be due to ectopic progenitor cells or occult tumor under imaging resolution.^9,10^ His presenting symptoms related to the locations of leptomeningeal polyradiculopathy and cervical myelopathy demonstrated by improvement in symptoms with steroids and medications for neuropathic pain. The methylation profiling was SHH-activated, with loss of one allele of Tp53 but no mutation, and histology showed a central area of desmoplastic/nodular morphology characterized by densely cellular sheets and single files of smaller nuclei interrupted by lucent, less cellular, well-circumscribed pale nodules of cells, consistent with medulloblastoma.

Robust prospective trials for adult treatment are lacking. The Packer protocol involves CSI with concurrent weekly vincristine, followed by maintenance cycles of cisplatin, lomustine, and vincristine.^11^ The rationale for using this protocol is to reduce the radiation dose, compensating with concomitant chemotherapy to achieve a synergistic effect and increase radiosensitivity.^12^ This approach was largely extrapolated from pediatric data. Adult patients tend to tolerate this protocol less; in one case series, only one of 10 adults completed all therapy.^13^ This can be managed by reducing concurrent vincristine dose or withholding it. On the other hand, around 60% of adults are SHH activated. This molecular profile allows for targeted therapy with agents like vismodegib.^14^

The patient had a very positive treatment response, but there are notable considerations and consequences that offer valuable lessons. In addition to the diagnostic challenges, he had some unique challenges immediately prior to and during treatment. He was of reproductive age, and he and his wife wanted to have children in the future, so prior to receiving radiation and chemotherapy, which are synergistically gonadotoxic and impair fertility, he underwent sperm banking.^15,16^ This was an important patient-centered aspect of treatment logistics and reminds clinicians to consider the reproductive health of their patients. Another aspect of the treatment plan was steroids due to structural compression and functional impairment via leptomeningeal congestion of cauda equina causing neuropathic symptoms. However, the long-term use of steroids led to fatigue from an endocrinopathy that did not meet criteria for adrenal insufficiency, and the duration of steroids was informed by monitoring and wanting to avoid the various adverse effects of steroids such as immunosuppression, osteoporosis, and peptic ulcer disease.^17^ In addition to this risk of a steroid-induced endocrinopathy, the more long-term risk of developing an endocrinopathy for this patient derives from undergoing whole brain radiation therapy (WBRT), which can result in hypopituitarism. In fact, in studies of survivors of childhood cancer, 66% of patients who received WBRT eventually developed some form of endocrinopathy.^18^ Finally, despite extensive leptomeningeal disease on imaging, CSF cytology testing did not identify cancerous cells, which is a known diagnostic limitation as the detection rate of CSF cytology on one sample ranges from 54% to 73%. This highlights the role of biopsy to make a definitive diagnosis.^19,20^

Learning points from this case include

- The importance of not anchoring on what prior evaluations may report and keeping differential diagnoses broad even if the presentation may be similar to a prior presentation, especially when the symptoms are nonspecific like headache.
- The importance of including spinal images in the context of unexplained increased ICP especially with other symptoms and signs localize to the spinal cord.
- Successful diagnosis required a critical examination of incongruencies between exam findings and prior diagnosis of IIH.
- It is important to recognize as well that the patient’s larger body habitus may have contributed to the diagnosis of IIH and led to bias with premature closure of alternative etiologies of headache.
- Recognizing that these exam findings would be inconsistent with IIH, prompted further imaging, which was then essential in determining the actual diagnosis.
- This case re-emphasizes of the limitation of CSF cytology to detect CNS malignancy.

## Conclusion

Leptomeningeal disease without a primary cancerous mass is rare. Leptomeningeal medulloblastoma without an index parenchymal lesion has not been previously described in adults, with just a few case reports in children, which contributed to this being a challenging case. While the diagnostic odyssey was protracted, the patient experienced fast and significant radiographic and clinical improvement to proton whole CSI followed by the Packer protocol indicating that this is an effective treatment for the rare condition of adult onset primary leptomeningeal medulloblastoma.

## Supplemental Material

sj-pdf-1-sco-10.1177_2050313X261459345 – Supplemental material for A diagnostic headache: A rare case of adult onset primary leptomeningeal medulloblastoma Supplemental material, sj-pdf-1-sco-10.1177_2050313X261459345 for A diagnostic headache: A rare case of adult onset primary leptomeningeal medulloblastoma by Sophia Raefsky, Neal Nathan, Meshari Alharthi, Jessica Bloom, Vanessa Goodwill, Kari Hird, Nikdokht Fari, William Ueng and Jessica Schulte in SAGE Open Medical Case Reports

## References

[1] Rozen TD. New daily persistent headache: clinical perspective. Headache 2011; 51(4): 641–649. 21457251 10.1111/j.1526-4610.2011.01871.x

[2] Louis DN. The 2021 WHO classification of tumors of the central nervous system: a summary. Neuro Oncol 2021; 23(8): 1231–1251. 34185076 10.1093/neuonc/noab106 PMC8328013

[3] Fabbro S Pegolo E Piccolo D , et al. Primary leptomeningeal medulloblastoma in adults: a diagnostic challenge – case report and systematic review. Asian J Neurosurg 2022; 17(4): 651–655. 36570761 10.1055/s-0042-1757726 PMC9771618

[4] Meister M Lin JJ Bach SE , et al. Medulloblastoma presenting as isolated leptomeningeal enhancement with no primary mass. Cureus 2022; 14(7): e26598. 10.7759/cureus.26598 PMC9352599 35936137

[5] Smoll NR. Relative survival of childhood and adult medulloblastomas and primitive neuroectodermal tumors (PNETs). Cancer 2012; 118(5): 1313–1322. 21837678 10.1002/cncr.26387

[6] Ala RT Yener G Özer E , et al. Adult spinal primary leptomeningeal medulloblastoma presenting as pseudotumour cerebri syndrome. Neuroophthalmology 2021; 45(3): 205–210. 34194127 10.1080/01658107.2020.1791191 PMC8210868

[7] Franceschi E Giannini C Furtner J , et al. Adult medulloblastoma: updates on current management and future perspectives. Cancers 2022; 14(15): 3708. 10.3390/cancers14153708 35954372 PMC9367316

[8] Yeo KK Margol AS Kennedy RJ , et al. Prognostic significance of molecular subgroups of medulloblastoma in young children receiving irradiation-sparing regimens. J Neurooncol 2019; 145(2): 375–383. 10.1007/s11060-019-03307-8 31621042 PMC7543681

[9] Russo C Scala MR Spennato P , et al. Primary leptomeningeal medulloblastoma: a case-based review. Childs Nerv Syst 2022; 38(3): 527–536. 10.1007/s00381-021-05435-x 35059784

[10] Mehta RI Cutler AR Lasky JL III , et al. “Primary” leptomeningeal medulloblastoma. Hum Pathol 2009; 40(11): 1661–1665. 10.1016/j.humpath.2009.04.024 19695677

[11] Packer RJ Siegel KR Sutton LN , et al. Efficacy of adjuvant chemotherapy for patients with poor-risk medulloblastoma: a preliminary report. Ann Neurol 1988; 24(4): 503–508. 10.1002/ana.410240405 3239953

[12] Crawford JR MacDonald TJ Packer RJ. Medulloblastoma in childhood: new biological advances. Lancet Neurol 2007; 6(12): 1073–1085. 10.1016/S1474-4422(07)70289-2 18031705

[13] Greenberg HS Chamberlain MC Glantz MJ , et al. Adult medulloblastoma: multiagent chemotherapy. Neuro Oncol 2001; 3(1): 29–34. 10.1093/neuonc/3.1.29 11305414 PMC1920599

[14] van den Bent MJ Geurts M French PJ , et al. Primary brain tumours in adults. Lancet 2023; 402(10412): 1564–1579. 10.1016/S0140-6736(23)01054-1 37738997

[15] Vakalopoulos I Dimou P Anagnostou I , et al. Impact of cancer and cancer treatment on male fertility. Hormones 2015; 14(4): 579–589. 10.14310/horm.2002.1620 26732148

[16] Kort JD Eisenberg ML Millheiser LS , et al. Fertility issues in cancer survivorship. CA Cancer J Clin 2014; 64(2): 118–134. 10.3322/caac.21205 24604743

[17] Nicolaides NC Pavlaki AN Maria Alexandra MA , et al. Glucocorticoid therapy and adrenal suppression. In: Feingold KR Anawalt B Blackman MR , et al. (eds.) Endotext (Internet). MDText.com, Inc., 2000, https://www.ncbi.nlm.nih.gov/books/NBK279156/

[18] Appelman-Dijkstra NM Kokshoorn NE Dekkers OM , et al. Pituitary dysfunction in adult patients after cranial radiotherapy: systematic review and meta-analysis. J Clin Endocrinol Metab 2011; 96(8): 2330–2340. 10.1210/jc.2011-0306 21613351 PMC3146793

[19] Wasserstrom WR Glass JP Posner JB. Diagnosis and treatment of leptomeningeal metastases from solid tumors: experience with 90 patients. Cancer 1982; 49(4): 759–772. https://doi.org/10.1002/1097-0142(19820215)49:4<759::aid-cncr2820490427>3.0.co;2-7 6895713 10.1002/1097-0142(19820215)49:4<759::aid-cncr2820490427>3.0.co;2-7

[20] van Oostenbrugge RJ Twijnstra A. Presenting features and value of diagnostic procedures in leptomeningeal metastases. Neurology 1999; 53(2): 382–385. 10.1212/wnl.53.2.382 10430430

