# Supplementary material for: A diagnostic headache: A rare case of adult onset primary leptomeningeal medulloblastoma
Source: SAGE Open Med Case Rep. 2026 Jun 29;14:2050313X261459345. doi: 10.1177/2050313X261459345 (PMC13319574; doi:10.1177/2050313X261459345)
Supplement: sj-pdf-1-sco-10.1177_2050313X261459345 – Supplemental material for A diagnostic headache: A rare case of adult onset primary leptomeningeal medulloblastoma [file sj-pdf-1-sco-10.1177_2050313X261459345.pdf]

CARE Checklist of information to include when writing a case report

CARE case report guidelines

(cc) BY-NC-ND

Topic	Item	Checklist item description	Reported on Line
Title	1	The diagnosis or intervention of primary focus followed by the words "case report"	1,2
Key Words	2	2 to 5 key words that identify diagnoses or interventions in this case report, including "case report"	36,37
Abstract (no references)	3a	Introduction: What is unique about this case and what does it add to the scientific literature?	8,10,11,12,13,14,15
	3b	Main symptoms and/or important clinical findings	18,19,20,21,22,23,24
	3c	The main diagnoses, therapeutic interventions, and outcomes	32,33,34
	3d	Conclusion—What is the main "take-away" lesson(s) from this case?	252
Introduction	4	One or two paragraphs summarizing why this case is unique (**may include references**)	65-147
Patient Information	5a	De-identified patient specific information.	yes
	5b	Primary concerns and symptoms of the patient.	Headache
	5c	Medical, family, and psycho-social history including relevant genetic information	negative
	5d	Relevant past interventions with outcomes	N/A
Clinical Findings	6	Describe significant physical examination (PE) and important clinical findings.	79-84
Timeline	7	Historical and current information from this episode of care organized as a timeline	65-84
Diagnostic Assessment	8a	Diagnostic testing (such as PE, laboratory testing, imaging, surveys).	82-109
	8b	Diagnostic challenges (such as access to testing, financial, or cultural)	N/A
	8c	Diagnosis (including other diagnoses considered)	102
	8d	Prognosis (such as staging in oncology) where applicable	194,195
Therapeutic Intervention	9a	Types of therapeutic intervention (such as pharmacologic, surgical, preventive, self-care)	110-117,200-202
	9b	Administration of therapeutic intervention (such as dosage, strength, duration)	as above
	9c	Changes in therapeutic intervention (with rationale)	N/A
Follow-up and Outcomes	10a	Clinician and patient-assessed outcomes (if available)	113
	10b	Important follow-up diagnostic and other test results	113
	10c	Intervention adherence and tolerability (How was this assessed?)	116
	10d	Adverse and unanticipated events	N/A
Discussion	11a	A scientific discussion of the strengths AND limitations associated with this case report	154
	11b	Discussion of the relevant medical literature **with references**.	154
	11c	The scientific rationale for any conclusions (including assessment of possible causes)	252
	11d	The primary "take-away" lessons of this case report (without references) in a one paragraph conclusion	231
Patient Perspective	12	The patient should share their perspective in one to two paragraphs on the treatment(s) they received	215-218
Informed Consent	13	Did the patient give informed consent? Please provide if requested	Yes ☑ No ☐
